# Prevalence of obesity in preschool Greek children, in relation to parental characteristics and region of residence

**DOI:** 10.1186/1471-2458-7-178

**Published:** 2007-07-25

**Authors:** Yiannis Manios, Vassiliki Costarelli, Maria Kolotourou, Katerina Kondakis, Chara Tzavara, George Moschonis

**Affiliations:** 1 Department of Nutrition & Dietetics, Harokopio University, 70 El. Venizelou Ave, 176 71 Kallithea, Athens, Greece

## Abstract

**Background:**

The aim of this retrospective cohort study was to record the prevalence of overweight and obesity in relation to parental education level, parental body mass index and region of residence, in preschool children in Greece.

**Methods:**

A total of 2374 children (1218 males and 1156 females) aged 1–5 years, stratified by parental educational level (Census 1999), were examined from 105 nurseries in five counties, from April 2003 to July 2004, Weight (kg) and height (cm) were obtained and BMI (kg/m^2^) was calculated. Both the US Centers for Disease Control (CDC) and the International Obesity Task Force (IOTF) methods were used to classify each child as "normal", "at risk of overweight" and "overweight". Parental demographic characteristics, such as age and educational level and parental anthropometrical data, such as stature and body weight, were also recorded with the use of a specifically designed questionnaire.

**Results:**

The overall estimates of at risk of overweight and overweight using the CDC method was 31.9%, 10.6 percentage points higher than the IOTF estimate of 21.3% and this difference was significant (p < 0.001). Children with one obese parent had 91% greater odds for being overweight compared to those with no obese parent, while the likelihood for being overweight was 2.38 times greater for children with two obese parents in the multivariate model.

**Conclusion:**

Both methods used to assess prevalence of obesity have demonstarted that a high percentage of the preschool children in our sample were overweight. Parental body mass index was also shown to be an obesity risk factor in very young children.

## Background

Overweight and obesity among preschool children is of great concern, because it may lead to long-term health consequences [1]. A number of recent studies have suggested that childhood obesity in most cases tracks into adulthood [2-4] and increases the risk of degenerative diseases later in life [5,6]. More specifically, it has been shown that 69% of children between the ages of 6 and 10 years with a Body Mass Index (BMI) greater than the 95th percentile will continue to be obese in their adult life [7]. In addition, conditions such as type 2 diabetes mellitus, hypercholesterolemia and hypertension, which were previously seen mainly in adults, are becoming more common among children as the prevalence of obesity increases [8].

Prevalence of childhood obesity is rising at an alarming rate worldwide [9-11]. However, limited infromation is available on prevalence of obesity and its contributing factors, in preschool children [12,13]. The few studies that have been published on overweight and obesity among preschool children, coming predominately for the USA and Canada, have demonstrated that a trend towards increasing weight is also occurring in this age group [12,14,15]. A similar trend was also recorded by the very small number of studies, conducted on the same issue, in Europe [16,17] and in particular southern Europe [18,19]. In the case of Greece, in spite of the long tradition on healthy eating and the relatively lower risk of degenerative diseases, the prevalence of obesity among adults and school children [20,21], has been increasing over the last decades.

In common with adulthood obesity, childhood obesity [22-25] seems to follow an ethnic, geographic and socio-economic distribution. However, very few studies have examined the possible impact of demographic and socioeconomic characteristics, in the risk of being overweight or obese, in the case of children under the age of 6 [12,19].

Recording and understanding the prevalence of obesity in young children and the social, geographic and cultural parameters related to the phenomenon, can facilitate the formation of effective public health intervention policies in counteracting childhood obesity. It is known that early prevention is more effective in managing the epidemic of obesity [26] in comparison to treating obesity later in life.

The aim of the study was to record the prevalence of overweight and obesity among pre-school children in Greece in relation to parental education level, parental body mass index and region of residence. The present study constitutes the first countywide representative report on the prevalence of overweight and obesity among preschool children in Greece.

## Methods

### Sampling

The study design is reported briefly since it has previously been presented elsewhere [27]. This retrospective cohort study involved Greek preschool children 12 to 60 months of age participating in the GENESIS study, which was carried out from April 2003 to July 2004.

A representative number of randomly selected public and private nurseries as well as day-care centers within municipalities in five counties (namely Attica, Aitoloakarnania, Thessalonica, Halkidiki and Helia) were invited to participate in the study. All nurseries invited to participate responded positively. Furthermore, an extended letter explaining the aims of the current study and a consent form was provided to each parent or guardian having a child in these nurseries. Those parents who agreed to participate in the study had to sign the consent form and provide their contact details. Signed parental consent forms were collected for 2518 children, aged 1 to 5 years old (Response rate 75%). From the total number of positive responses complete data became available for 2374 children with participation rate varying from 54% to 95%, reaching the highest rate in rural areas and the lowest one in urban areas.

These counties are scattered over the Greek dominion while their overall local population comprises about 70% of the total Greek population (Census 1999). Among the total number of nursery schools studied (n = 105 out of a total of 313), 63 were in Attica (out of 121), 8 were in Thessalonica (out of 108), 12 were in Halkidiki (out of 13), 22 were in Aitoloakarnania (out of 37) and 7 were in Helia (out of 34). The sampling of the nurseries was random, multistage and stratified by the total population of children, according to data provided by the National Statistical Service of Greece (Census 1999).

The participating regions were grouped based on their population in "Large Urban Areas", with a population size greater than 1,000,000; "Urban Areas" with a population size ranging from 10,000 to 100,000; and "Rural Areas and Small Towns" with a population size less than 10,000 inhabitants. The aforementioned classification stems from a particularity of the population distribution in Greece, according to which there are plenty of "Rural Areas and Small Towns", as well as "Urban" areas but only two "Large Urban" areas of approximately 1,200,000 and 4,500,000 citizens, respectively. The detailed description of the study population by region of residence is described in table 1.

**Table 1:** Description of the study population by region of residence

	Boys		Girls	
	n	%	n	%

Region of Residence				
Rural Areas & Small Towns	261	21.4	247	21.4
Urban Areas	281	23.1	252	21.8
Large Urban Areas	676	55.5	657	56.8
Total	1218		1156	

Approval to conduct the study was granted by the Ethical Committee of Harokopio University of Athens and by all municipalities invited to participate in the study.

### Anthropometrical measurements

All study sites used the same measuring equipment and procedures. The instruments needed to be accurate and precise, yet portable enough to be carried to the nursery schools, where the measurements took place. Measurements were taken and recorded by two well-trained team members, which are referred as "leading" and "assisting" observer, respectively. The role of the "assisting" observer was to help position the child correctly to the instruments, while the "leading" observer recorded the measurements.

Body weight was recorded to the nearest 10 g with the use of a Seca digital scale and with subjects standing without shoes in the minimum clothing possible, i.e. underwear. Recumbent length was measured for all subjects to the nearest 0.1 cm with a portable measuring wooden board that had a stationary head piece, a sliding vertical foot piece and a horizontal back piece with a measure tape mounted on it. Further to recumbent length, standing height was also measured to the nearest 0.1 cm in children older than two years of age, with the use of a commercial stadiometer (Leicester Height Measure). The measurement of height was conducted without shoes and with children keeping their shoulders in a relaxed position, their arms hanging freely and with their head aligned in Frankfurt plane. Body Mass Index (BMI) was calculated by dividing weight (Kg) with standing height squared (m^2^).

### Definition of overweight

In order to make the data most useful for comparison, and to contribute to the understanding of international standard definitions of overweight and obesity, both the US Centers for Disease Control (CDC) [28] and the International Obesity Task Force (IOTF) [29] methods were used to classify each child as "normal", "at risk of overweight" and "overweight".

More specifically the Nutstat module of EpiInfo [46] was used to determine the age and sex-specific percentiles for weight, length and BMI, according to the Centers for Disease Control (CDC) 2000 Growth Charts [47]. Using the CDC weight-for-length growth charts children up to 24 months of age were classified as underweight (≤5^th ^percentile) and overweight (≥95^th ^percentile). For children older than 24 months, the CDC BMI-for-age growth charts were used for their categorization as underweight (≤5^th ^percentile), at risk of overweight (85^th^–95^th ^percentile) and overweight (≥95^th ^percentile).

The BMI cut-off criteria adopted by the IOTF of childhood equivalents of overweight were also used in children over the age of two [29].

### Additional information obtained from parents

During the morning interview at the nursery, additional information was obtained from the guardians with respect to: (a) parental demographic characteristics, such as age and educational level; (b) parental anthropometrical data, such as stature and body weight, using a specially designed questionnaire. The reliability of the self reported questionnaire administered to the parents was found to be sufficient, with a cronbach-α coefficient equal to 0.78.

### Statistical analysis

All variables are reported categorically. For the comparison of the proportion of children categorized as at risk of overweight or overweight according to the CDC and IOTF methods z-tests were used. In order to control for multiple testing a significance level ≤0.001 was set. Chi-square tests were used to evaluate the association of being overweight with variables under investigation. P values for trend in prevalence of overweight children according to parental weight status, were computed. Data were modeled using multiple logistic regression analysis. Odds ratios with 95% confidence intervals were computed from the results of the logistic regression analyses. All p values reported are two-tailed. Statistical significance was set at 0.05 and analyses were conducted using STATA statistical software (version 6.0). Finally, demographics were treated as random effects since the data were collected by random sampling.

## Results

The prevalence by the CDC method of at risk of overweight and overweight was 16.3 and 16% in boys and 16.2 and 15.5% in girls, respectively. The prevalence by the IOTF method of at risk of overweight and overweight was 12.9 and 6.2% in boys and 15.5 and 8.1% in girls, respectively. Table 2 presents the prevalence of at risk of overweight and overweight children, by age group and sex according to both methods (CDC and IOTF). The proportion of children categorized as overweight using the CDC method was significant higher both for boys and girls. The overall estimates of at risk of overweight and overweight using the CDC method was 31.9%, 10.6 percentage points higher than the IOTF estimate of 21.3% and this difference was significant (p < 0.001). Both boys and girls were 1.72 times more likely to be classified as at risk of overweight or overweight using the CDC method as compared to the IOTF method. Furthermore, both boys and girls were 2.42 times more likely to be classified as overweight using the CDC method as compared to the IOTF method. A boy was 2.86 times more likely to be classified as overweight by the CDC method than by the IOTF method, while a girl was 2.08 times more likely to be classified as overweight by the CDC method than by the IOTF method.

**Table 2:** Prevalence (%) of obesity by gender and age

	Weight status of children					
	CDC	IOTF	CDC	IOTF	CDC	IOTF
	Normal weight*		At risk for overweight		Overweight	

Males (n = 1218)						
Age group (years)						
1–2 (n = 100)	83.5				16.5	-
2–3 (n = 274)	66.1	82.3‡	20.1	13.8	13.8	3.9‡
3–4 (n = 488)	65.1	78.0‡	18.4	15.3	16.5	6.7‡
4–5 (n = 356)	68.5	77.9	14.8	12.9	16.7	9.2
All ages	67.8	80.8‡	16.3	12.9	16.0	6.2‡

Females (n = 1156)						
Age group (years)						
1–2 (n = 107)	88.6				11.4	-
2–3 (n = 226)	67.1	77.5	18.3	15.8	14.6	6.7
3–4 (n = 434)	68.5	74.2	15.5	17.8	16.0	8.0‡
4–5 (n = 389)	64.0	72.3	19.6	16.9	16.4	10.9
All ages	68.3	76.4‡	16.2	15.5	15.5	8.1‡

*For CDC classification normal weight or underweight

‡p < 0.001 for the comparison of proportions by the two methods

US Centers for Disease Control (CDC)

International Obesity Task Force (IOTF)

**Figure 1:**
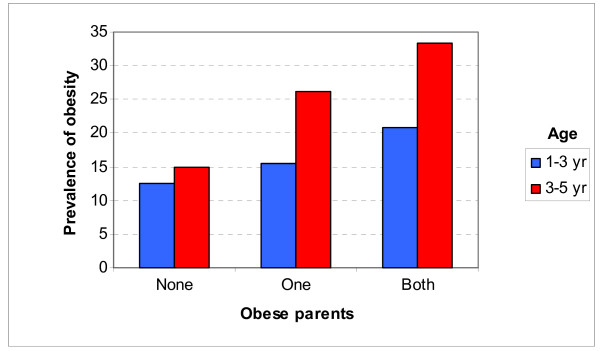
Prevalence of obesity by age group and parents' weight status.

Table 3 illustrates the prevalence of being overweight, and of being overweight or at risk for overweight (CDC cut off points), for age groups 1–3 and 3–5 years, by region, gender, mother' s age, educational level of the mother and the father and weight status of the parents. The prevalence of being overweight in both age groups was significant greater for children with one or two obese parents (figure 1) and increases as the number of obese parents increases (p-value for trend <0.001 in both sexes). Furthermore, The prevalence of being overweight or at risk for overweight in age group 3 to 5 years was significantly greater for children with one or two obese parents. When multiple logistic regression analysis was conducted it was found that children with one or two obese parents had 1.96 times greater odds for being overweight compared to those with no obese parent and 1.70 times greater odds for being overweight or at risk for overweight, after adjusting for region, age group, gender, age of the mother and educational level of the mother. Additionally, children with one obese parent had 91% greater odds for being overweight compared to those with no obese parent, while the likelihood for being overweight was 2.38 times greater for children with two obese parents in the multivariate model (Table 4).

**Table 3:** Socio-economic status, parental characteristics and prevalence of overweight in children aged 1–3 and 3–5 years.

	(1–3 years)		(3–5 years)		(1–3 years)		(3–5 years)	
	% overweight	P†	% overweight	P†	% At risk for overweight or overweight	P†	% At risk for overweight or overweight	P†

Region								
*Large urban*	12.3	0.413	16.4	0.664	27.3	0.214	34.3	0.560
*urban*	17.0		17.8		34.6		34.4	
*rural/small towns*	13.8		18.7		27.0		37.7	
Gender								
*Male*	14.5	0.731	16.6	0.814	29.9	0.612	34.7	0.769
*Female*	13.6		16.2		28.1		35.4	
Mothers' age								
*<30*	11.9	0.641	18.9	0.662	28.3		36.3	
*31–36*	15.0		16.6		29.2	0.950	34.6	0.872
*>36*	14.6		17.0		29.8		34.7	
Educational level of the mother (years)								
*≤9*	14.8	0.986	22.5	0.198	27.8	0.949	43.5	0.062
*10–14*	14.2		17.0		29.1		35.1	
*>14*	13.9		16.2		29.8		33.2	
Educational level of the father (years)								
*≤9*	13.5	0.503	18.0	0.951	30.2		37.4	
*10–14*	15.7		17.1		29.8	0.901	35.5	0.628
*>14*	12.1		17.2		28.1		33.8	
Obesity of the parents None								
One/Both	12.5	0.034	14.9	<0.001	27.7	0.116	32.1	<0.001
	20.2		27.0		35.1		47.0	

† P value for χ^2 ^test

## Discussion

The current study is the first to report on the prevalence of overweight on a representative population sample of Greek preschoolers. It has revealed a high prevalence of obesity in this age group and a higher risk of being obese with increased parental overweight. In order to enhance the understanding of international standard definitions of overweight and obesity and to make the data most useful for comparison, both the US Centers for Disease Control (CDC) [28] and the International Obesity Task Force (IOTF) [29] methods were used to classify each child as "normal", "at risk of overweight" and "overweight".

The study has shown that both methods have resulted in high estimates of overweight and obesity overall, there were however, a number of differences between the two methods (Table 2). The overall estimates of at risk of overweight and overweight using the CDC method was 31.9%, 10.6 percentage points higher than the IOTF estimate of 21.3% and this difference was significant (p < 0.001). Most importantly the data indicates that boys were 2.86 times more likely to be classified as overweight by the CDC method than by the IOTF method, while girls was 2.08 times more likely to be classified as overweight by the CDC method than by the IOTF method. The estimate of overweight and risk of overweight using the CDC method was considerably higher than the IOTF estimate which is in accordance with other published data on older children [30,31]. As reported by Vidal *et al*, 2006, the difference between the sensitivity of the above two methods to detect obesity, is even larger when evaluating children under the age of 5, which is in accordance with the findings of the present study [13]. As a result, it is important to stress the need to ensure that the same cut off reference values have been used to define overweight and obesity, when comparing and contrasting studies on prevalence of obesity in children and in particular preschoolers. There were no statistical differences in the prevalence of obesity between sexes, in our study.

The prevalence of overweight and obesity recorded in this study (table 2) is comparable to that reported for preschool children in other developed countries, where high rates of childhood obesity co-exist, regardless of the method used to determine obesity. Ogden and colleagues, in the United States, found that the prevalence of obesity (>95th percentile) was 10.4% among children 2–5 years old [32] whereas more recently Whitaker et al. 2006, has reported that 14.8% of caucasian preschoolers were classified as obese (CDC cut off points) [12]. The equivelant prevalence of obesity ("overweight") in our sample was higher reaching 16% for boys and 15.5% for girls (table 2). In Canada, a similar study in preschool children has reported that 7.8% of boys and 8.2% of girls were obese, using the IOTF cut off points, whereas the equivelant percentages from our sample were very comperable at 6.2% and 8.1% repsectively [14]. However, the prevalence of obesity in 2–3 year old children using the IOFT cut off points recorded by the current study, was 3.9% for boys and 6.7% for girls. These finding are considerably higher than the prevalence recorded by a recent similar study conducted in Cyprus where the prevalence of obesity (IOTF definition) was 1.3% in 2 y olds [19].

One of the aims of this study was to determine the age at which overweight and obesity develops in toddlers. The data revealed that 16.5% of boys and 11.4% of girls between 1–2 years old were classified as "overweight" (CDC cut off points/I.O.T.F are only valid after the age of 2 years old). It is important to note that the reported high prevalence of obesity in very young children, indicates an increased risk for even higher rates of obesity in adolescence and adulthood, in the near future, exceeding those currently reported.

The environmental and social changes that promote a sedentary lifestyle and an increased consumption of energy dense foods, have been identified as major causes of obesity in childhood [33,34]. Sociodemographic and parental characteristics have also been implicated [22,23]. This study was also set out to investigate possible links between obesity in very young children with parental body mass index, parental education level and region of residence.

The findings indicate that high parental weight increased the risk of being "overweight" or "at risk of overweight" in this sample. More precisely, the prevalence of being overweight in both age groups was significantly greater for children with one or two obese parents (figure 1) and increases as the number of obese parents increases (p-value for trend <0.001 in both sexes). This trend of increased risk of overweight with increasing parental overweight is in line with other published data [35,36]. This effect of parental obesity on children's risk for increased adiposity is one of the most consistent findings in the controversial field of obesity [36,37]: Some researchers argue that children adopt their parents' eating habits as a result of environmental exposure rather than the heredity of "food choice genes", although it is unquestionable that some of this resemblance is attributed to genetic similarities [38,39].

In accordance to other studies [40,41], region of residence was not found to influence the risk of overweight, although there is some indication of an increased prevalence of childhood obesity in urban compared to rural areas in Greece [42]. It was reported that in the case of 6–18 year old children, different sosio-econimic status (SES) groups are at different risk of obesity and the relationship between SES factors and obesity varies across countries [12,40]. A recent study by Savva *et al*., 2005 conducted in Cyprus in preschool children has reported that overweight and obesity prevalence was higher in children living in rural areas (16.1%) in comparison to urban areas (12.8%; *P *= 0.046) [19].

Parental SES has been suggested as a potential risk factor for childhood obesity [43,44]. Lamerz and collegues [22] has reported a strong relationship between parental years of education and childhood obesity. Simlar findings where also reported by Lien in adolescents [45]. However, in our study maternal and paternal educational level, an indirect indicator of SES, was not found to influence the risk of overweight. It is likely that parental education is not related to the presence of overweight and obesity in very young children and only becames a causative factor in later years. It can also be postulated that in the case of very young children, there is not yet enough time for possible non genetic parameters such as the social and physical environment to have a significant impact on the prevalence of obesity.

## Conclusion

In conclusion, the prevalence of overweight in Greek preschoolers is very high and is strongly related to parental overweight. Given the fact that Greece is one of the European countries with the highest prevalence of childhood obesity [34], the above findings should guide the public health policy to target risk of obesity and overweight with appropriate intervention strategies early in life.

## Competing interests

The author(s) declare that they have no competing interests.

## Authors' contributions

YM was in charge of study design, data collection and analysis; MK, VC, KK and GM carried out the field work and data entry; CT performed the statistical analysis; YM, VC, MK, KK, CT and GM contributed in data interpretation and writing the manuscript.

**Table 4:** Results of multiple logistic regression models: adjusted odds ratios – 95% confidence intervals for being overweight and at risk for overweight or overweight

	*Overweight*		*At risk for overweight or overweight*	
	OR (95% CI)†	P	OR (95% CI)†	P

Model 1				
Obesity of the parents				
None	Reference		Reference	
One/Both	1.96 (1.48–2.59)	<0.001	1.70(1.34–2.14)	<0.001

Model 2				
Obesity of the parents				
None	Reference		Reference	
One	1.91 (1.42–2.56)	<0.001	1.57 (0.87–2.81)	0.134
Both	2.38(1.25–4.53)	0.008	1.72(1.34–2.19)	<0.001

† adjusted for region, age group, gender, age of the mother, educational level of the mother

## Pre-publication history

The pre-publication history for this paper can be accessed here:


